# Identification and characterization of rye genes not expressed in allohexaploid triticale

**DOI:** 10.1186/s12864-015-1480-x

**Published:** 2015-04-10

**Authors:** Hala Badr Khalil, Mohammad-Reza Ehdaeivand, Yong Xu, André Laroche, Patrick J Gulick

**Affiliations:** Department of Biology, Concordia University, 7141 Sherbrooke W., Montreal, Quebec H4B 1R6 Canada; Agriculture and Agri-Food Canada, Lethbridge Research Centre, Lethbridge, AB T1J 4B1 Canada; Present addresses: Department of Genetics, Faculty of Agriculture, Ain-Shams University, Shoubra El-khema, Cairo, Egypt; Agriculture and Agri-Food Canada, Pacific Agri-Food Research Center, Summerland, BC V0H 1Z0 Canada

**Keywords:** Allopolyploidization, Gene repression, Gene deletion, Gene silencing, Triticale, High-throughput DNA sequencing, Tissue-specific expression

## Abstract

**Background:**

One of the most important evolutionary processes in plants is polyploidization. The combination of two or more genomes in one organism often initially leads to changes in gene expression and extensive genomic reorganization, compared to the parental species. Hexaploid triticale (x *Triticosecale*) is a synthetic hybrid crop species generated by crosses between *T. turgidum* and *Secale cereale*. Because triticale is a recent synthetic polyploid it is an important model for studying genome evolution following polyploidization. Molecular studies have demonstrated that genomic sequence changes, consisting of sequence elimination or loss of expression of genes from the rye genome, are common in triticale. High-throughput DNA sequencing allows a large number of genes to be surveyed, and transcripts from the different homeologous copies of the genes that have high sequence similarity can be better distinguished than hybridization methods previously employed.

**Results:**

The expression levels of 23,503 rye cDNA reference contigs were analyzed in 454-cDNA libraries obtained from anther, root and stem from both triticale and rye, as well as in five 454-cDNA data sets created from triticale seedling shoot, ovary, stigma, pollen and seed tissues to identify the classes of rye genes silenced or absent in the recent synthetic hexaploid triticale. Comparisons between diploid rye and hexaploid triticale detected 112 rye cDNA contigs (~0.5%) that were totally undetected by expression analysis in all triticale tissues, although their expression was relatively high in rye tissues. Non-expressed rye genes were found to be strikingly less similar to their closest BLASTN matches in the wheat genome or in the other *Triticum* genomes than a test set of 200 random rye genes. Genes that were not detected in the RNA-seq data were further characterized by testing for their presence in the triticale genome by PCR using genomic DNA as a template.

**Conclusion:**

Genes with low similarity between rye sequences and their closest matches in the *Triticum* genome have a higher probability to be repressed or absent in the allopolyploid genome.

**Electronic supplementary material:**

The online version of this article (doi:10.1186/s12864-015-1480-x) contains supplementary material, which is available to authorized users.

## Background

The cause and mechanisms of the striking alteration of plant genomes after allopolyploidization has been a central question in allopolyploid genome evolution. Plants, unlike animals, are relatively tolerant to interspecific genome hybridization and chromosome duplication, and polyploidy is relatively common among plant species. The studies of paleopolyploids indicate the diploidization process involves major genome rearrangements including chromosome loss [1], reduction in chromosome number by various forms of chromosome fusion and rearrangements, gene loss [2], changes of gene expression [3], and in some cases genome expansion [4]. More recent polyploids such as the tetraploid *Triticum turgidum*, and the hexaploid *Triticum aestivum*, thought to have formed 0.5 MYA, and 0.01 MYA, respectively [5], and polyploid *Brassica* species, thought to have formed 5,000-10,000 YA, maintain polyploid chromosome numbers but have diploid chromosome pairing patterns during meiosis. The genomes maintain synteny, but nevertheless undergo gene loss [6,7], gene silencing [8], inversion [9] and translocation events [10].

Although the mechanisms of gene silencing and elimination are still unknown, several studies have found that these changes occur rapidly and occur more frequently in one of the two parental genomes of an allotetraploid as reported for *Triticum* [8,11], *Tragopogon* [12,13] and *Gossypium* [14]. The preferential control of traits by the genes from one parental genome, is apparently not random in allopolyploids and natural selection for balanced gene dosage effects has a strong impact on this process [15]. Phenotypic comparisons of allotetraploid and allohexaploid wheat, and their diploid parents indicated that genes controlling traits related to domestication such as autogamy, non-brittle spike, free-threshing glumes, and large kernel size are predominately controlled by genes of the A genome. In contrast, the B and D genomes preferentially control biotic and abiotic stress-regulated gene expression [reviewed in 11 and 15].

A significant degree of genome alteration occurs during allopolyploidizations. The amount of total nuclear DNA assayed in both natural and newly generated wheat polyploids was found to be 2-10% less than the sum of the amount of DNA of their parents [16,17]. The synthetic allohexaploid triticale has a genome structure similar to hexaploid bread wheat except that it has rye as one of its progenitors instead of the D genome donor *Aegilops tauschii*. It was first developed in the late 19th century, and is derived from a cross between tetraploid wheat, *T. turgidum*, and *Secale cereale*, and contains the A, B, and R genomes [18]. Current triticale lines originate from more recent intergeneric crosses beginning in the 1960’s. Triticale is an important model for studying the rapid changes that occur subsequent to polyploidization involving genomic remodeling and changes in gene expression. The hexaploid genome of triticale was found to have a high degree of DNA reduction with measurements of DNA loss in the range of 22-30% [19,20]. Most losses have been documented to occur in the first generations, up to and including the 5^th^generation [21]. This high degree of change from this wide cross makes triticale an important model for characterizing these rapid changes at both the level of gene expression and genomic restructuring following allopolyploidization.

Molecular techniques have been developed to facilitate the global estimation of homeologous gene silencing in both natural and synthetic allopolyploids. The implementation of cDNA-AFLP, a qualitative method employed to study transcriptional changes, detected absence of expression of homeologous genes in several synthetic allopolyploids. These studies found gene silencing for approximately 5% of genes in allopolyploid cotton [14], between 1% and 5% of genes in allotetraploid wheat [8], 0.4% of genes in Arabidopsis [22], and 9% and up to 30% of genes in octoploid and hexaploid triticale, respectively [23]. In addition, these studies detected changes of tissue-specific gene expression of many genes, a phenomenon referred to as subfunctionalization [14]. Comparative gene expression studies by microarray analysis revealed that 19% of the genes analyzed in wheat showed more than a 5 fold difference in expression between homeologous gene copies [24]. Microarrays and cDNA-AFLP analyses are highly sensitive tools used in several molecular studies to detect changes of gene expressions in polyploids; however, there is experimental variability arising from PCR in the analysis of a large number of bands in AFLP, and from the variability of fluorescent signals in microarrays. There is also a paucity of probes that can distinguish between highly similar homeologous gene copies on microarrays. Estimating gene expression using second generation high-throughput cDNA sequencing techniques offers the advantage of increasing the accuracy of transcript identification directly from the sequence rather than by DNA or RNA hybridization. Here, we investigate the impact of allopolyploidization on the rye coding sequences in the triticale transcriptome at a high level of resolution using second generation Roche 454-cDNA sequencing technology. The next generation sequencing data is a particularly important advancement for analysis of polyploids such as wheat or triticale, since homeologous genes have very high sequence similarity and often cannot readily be distinguished by hybridization techniques. A comparison of the transcription level of 23,503 rye reference contig assemblies between triticale and rye tissues has been used to detect and characterize the classes of rye genes prone to be either silenced or absent in the allopolyploid.

## Methods

### Rye, triticale and wheat growth conditions

Seeds of rye (*S. cereale*, 2n = 2x, RR, cv. Muskateer and Prima), triticale, (x *Triticosecale* 2n = 6x, AABBRR,, cv. AC Certa and Pika), as well as the spring and winter near-isogenic lines (NIL) of Anza bread wheat, (*T. aestivum*, 2n = 6x, AABBDD), were germinated in 20 cm pots containing equal volumes of peat moss, vermiculite, and black earth, and grown under 16 h light and 8 h dark at 22°C. After fifteen days, the seedling shoots and the roots of the two cultivars of each species were collected individually, frozen immediately in liquid nitrogen, and stored at −80°C. Floral tissues from triticale and rye were harvested from plants grown as described by Tran *et al.* [25], and samples were taken at different Zadoks developmental stages [26].

### Rye reference cDNA assemblies not detected by RNA-seq in triticale tissues

A rye gene reference set of 23,503 cDNA contigs was assembled from rye 454-cDNAs and was used to study their expression in triticale and rye tissue sets. A total of 6,674,733 cDNAs from triticale, and 1,999,453 cDNAs from rye, were derived from tissue-specific triticale libraries from seedling shoots, stem, root, stigma, anther and pollen, and from rye tissue-specific 454-cDNA libraries from stem, root and anther as previously described [27]. In addition, similarly constructed libraries were made from triticale seeds and triticale ovaries, and PCR amplified libraries were constructed from rye pistils. Root cDNA libraries from hydroponically grown plants were sequenced using the same Roche 454 GS FLX Titanium technology at Genome Quebec Innovation Centre, Montreal, PQ, Canada, described in [27]. The library sizes ranged from approximately 120,000 reads to over 1.2 M. Quality control analysis of triticale and rye 454-cDNAs was carried out by deleting continuous nucleotides with Phred scores less than 15 from the ends of reads, and masking internal nucleotides with Phred scores less than 20 with N’s using the FASTQ quality trimmer and FASTQ masker tools [28] available by free browser-based access through the Galaxy server from Penn State and Emory University [29]. The high quality 454-cDNAs obtained from each triticale and rye tissue were aligned to rye reference assemblies using the BWA-SW algorithm aligner [30] with default parameters, except mismatching penalty and z-best heuristics were set at 10, and 100, respectively. The transcripts uniquely mapped to each rye reference sequence were selected and counted. The expression of each rye contig in the reference assemblies was normalized based on the depth of each library and the length of each rye reference sequence using the reads per kilobase per million reads (RPKM) normalization units. Initially, all rye contigs were compared to the triticale reads to detect rye genes that were not expressed in triticale. A subset of more highly expressed rye reference sequences with a minimum level of expression of at least 10 transcripts in any rye tissue-specific library and not detected in all triticale libraries were selected for further analysis.

### Identifying most similar *Triticum* and *Aegilops* sequences corresponding to rye genes not detected in triticale tissues

The rye genes whose expression was not detected in triticale and a control set of 200 rye reference cDNA sequences were used to identify the most similar genes in the A and B genomes of *T. aestivum,* in the IWGSC-WSS survey sequence repository [31]. In addition, they were also used to identify the most similar sequences in *T. urartu* and *T. tauschii*, the A and D genome progenitors, using the *T. urartu* and *T. tauschii* genome scaffolds in GenBank (GB: AOTI00000000 and GB: AOCO010000000, respectively) through a BLASTN search. The most similar gene sequences were also searched for in the *T. aestivum* GenBank EST database (Release, May 4, 2012). The most similar A, B and D gene copies in all the databases that had an alignment block of at least 100 nt were selected. When the cDNA matched an accession with multiple blocks of alignment, e.g. from multiple exons, the percent identities between the most similar A, B and D hits to rye sequences were calculated based on the total length of the alignment blocks of each hit.

### Gene ontologies for rye-specific non-expressed sequences

The selected set of genes that were highly expressed in rye and which were not found to be expressed in the eight triticale tissues was further characterized by their ontologies. They were compared to GenBank databases using the BLAST2GO workstation [32]. Functional annotations were taken by sequence comparison to the GenBank non-redundant protein database using BLASTX with a threshold E-value of e^−10^.

### Screening for rye gene presence and absence

Ten non-expressed rye genes were selected for further characterization by assaying for their presence in the triticale genome by PCR using genomic DNA as a template. Ten pairs of rye gene-specific primers (Additional file 1: Table S1) were employed to screen genomic DNA for the presence/absence of these sequences using genomic DNA from two triticale cultivars, Pika and AC Certa. Rye cultivars, Musketeer and Prima, and the NIL of the wheat cultivar Anza, were used as positive and negative controls for the presence of DNA sequences. The genomic DNAs were extracted from one week old seedlings using a CTAB protocol [33]. PCR amplification was performed with *Taq* polymerase using 2 mM MgCl2, 0.2 mM dNTP, 1X Taq buffer and 10 μM of each primer under the following conditions: 95°C for 4 min; followed by 40 cycles of 30 sec at 94°C, 40 sec at a temperature between 54°-61°C depending on the specific primers used, and 1 min at 72°C; these cycles were followed by 12 min at 72°C.

### Validation of non-expressed rye-specific transcripts using RT-PCR

To validate the lack of expression in triticale of genes from the rye sub-genome, RT-PCR was performed by amplifying a selected set of rye coding sequences. Total RNA was extracted from the roots and shoots of seedlings of rye, triticale, and wheat cultivars using TRIzol reagent (Invitrogen) according to manufacturer’s instructions. Reverse transcription reactions included: 1 μg RNA, 50 μM oligo dT primer, 1 μl RNAse inhibitor, and 5 μl 5X RT buffer, brought up to a 25 μl total volume in DEPC-treated water. The reaction mixture was incubated at room temperature for 2 min, and 1 μl M MuLV reverse transcriptase New England Biolabs (200 units/ml) was added to each tube, mixed, and held at room temperature for 10 min, incubated at 42°C for 50 min and terminated at 70°C for 15 min. The same rye oligo nucleotide primers used for testing gene deletion were employed for RT-PCR and reactions were carried out using rye, triticale and wheat first strand cDNAs. PCR amplifications with *Taq* polymerase were performed under the following conditions: 95°C for 2 min, followed by 35 cycles of 30 sec at 94°C, 40 sec at 54-61°C, 1 min at 72°C; these were followed by 12 min at 72°C.

### Statistical analysis

Chi Squared (χ2) contingency tests were used to test the null hypothesis that there were no differences in sequence similarity between rye genes not detected in triticale and random control rye genes, and their closest match in the wheat IWGSC and EST databases. χ2 contingency tests were also used to test the hypothesis that there were no differences between the rye genes not detected in triticale and random rye genes in their degree of similarity to their highest match in the diploid genomes of *T. urartu* and *T. tauschii.*

## Results and discussion

### Rye genes not detected by RNA-seq in triticale

Screening a set of 23,503 rye reference contig sequences derived from Roche 454 cDNA reads with high-throughput RNA-seq profiling data sets from diploid rye for expression in hexaploid triticale, revealed that 465 transcripts, or approximately 2% of rye genes, were not detected in triticale. The expression of these genes was analyzed in 454-cDNA libraries obtained from anther, root and stem of both triticale and rye as well as from five triticale data sets created from ovary, pollen, seed, seedling shoot and stigma (Additional file 2: Figure S1). Further analysis was narrowed to a subset of genes that had relatively high expression in rye, namely 112 rye genes, i.e. approximately 0.5% of the genes in the reference set, that were represented by at least 10 transcripts in at least one of the rye tissues but which were not detected among the 6,674,733 triticale cDNA reads. Based on the level of expression in rye and the depth of the libraries for triticale (>10 reads; see Additional file 3: Table S3 and Additional file 4: Table S4), the probability of not detecting a rye transcript in triticale is <0.003, if the level of gene expression was 1/3 of its level of expression in rye.

### Rye sequence comparison to *Triticum* and *Aegilops* databases

**Figure 1:**
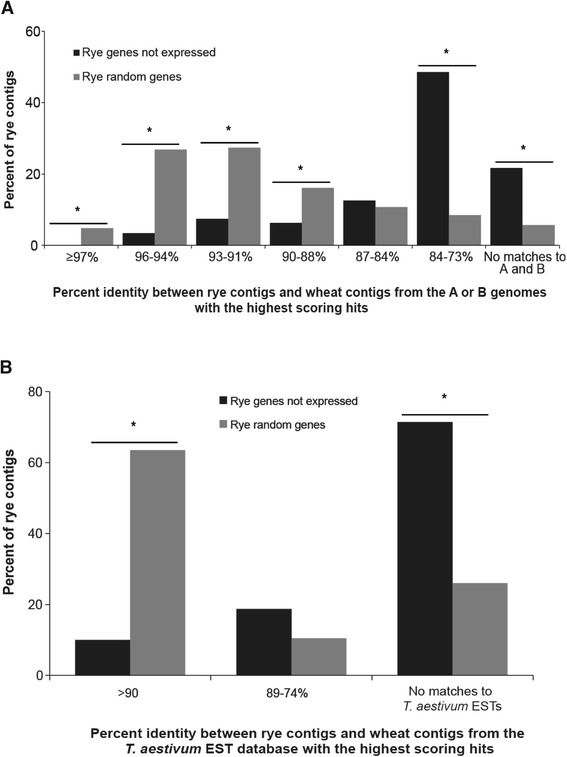
**Comparisons of rye genes not expressed in triticale and 200 random selected rye genes to** ***T. aestivum*** **databases. (A)** The majority of the 112 rye genes not expressed in triticale has lower sequence identity to wheat genome sequences in the IWGSC-WSS database (black bars) than to the control set consisting of 200 rye random sequences (gray bars). **(B)** Approximately 10% of rye genes not expressed in triticale had more than 90% sequence identity to the *T. aestivum* GenBank EST database (black bars), while 64% of the control set of 200 rye random sequences has high sequence identity to wheat ESTs (gray bars). χ2 contingency tests were carried out to test the if the number of rye genes not expressed triticale was significantly different from the number of random control rye genes in each class of percent identity of hits against the wheat A and B genomic sequences and the wheat EST databases, respectively. *marks significant differences with p < 0.01 in the number of contigs from the non-expressed rye genes and random rye genes in each class of percent identities between the rye contigs and wheat contigs.

To investigate the potential relationship between the triticale genes from the rye sub-genome that were not expressed in the allopolyploid, the corresponding rye contigs were compared to the genome sequence assemblies from *T. aestivum*. The comparison revealed the striking feature that most of the rye genes silenced in triticale mRNA pools did not have a homolog with sequence similarity ≥90% in *T. aestivum,* indicating that they possibly do not have a closely related homeologous copy in triticale. The distribution of the percent identity between the subset of 112 non-expressed rye genes to their closest matches in the A and B genomes of *T. aestivum* in the IWGSC-WSS database was significantly lower than a parallel comparison of a set of 200 randomly selected rye genes. More than 50% of rye sequences not expressed in triticale had a sequence similarity level between 73-84% with its most similar match in the wheat sequence assemblies (Figure 1A, Additional file 5: Table S5). The average DNA sequence identity between rye genes not detected in triticale and their most similar contigs in the A and B genomes in *T. aestivum* was only 81%. This degree of identity was significantly lower than the global average of 91% identity between the set of 200 randomly selected rye genes and their best matches in the A and B genomes of *T. aestivum* (Figure 1A). There appears to be a bias for silencing or deletion of rye genes that have low similarity to their most closely matched sequence in the *Triticum* genome. Previous studies of well-characterized gene families in the triticale found sequence identity between the ten members of the caleosin gene family in rye and their orthologs in *T. aestivum* to range between 99% to 90% within the coding region [27]. This degree of identity is similar to that among homeologous caleosin gene copies from the A, B and D genomes of *T. aestivum* and their orthologs in *Hordeum vulgare*, another member of the Triticeae [27]. Members of the α-tubulin gene family, as well as the Acc-1 and Acc-2 genes, also show high levels of similarity between homeologous gene copies within *T. aestivum* [5,34].

We further investigated the relationship between the rye genes not expressed in triticale to sequences from *T. aestivum*. Reciprocal comparisons were carried out by first comparing the non-expressed rye genes to the wheat EST sequences in GenBank, and then comparing the resulting highest scoring wheat hits back to the rye gene reference set, the 23,503 rye assemblies, to determine if this reciprocal comparison would result in matches that were more similar than that of the original comparison. The first comparison to the wheat EST database indicated that 90% of the non-expressed rye genes in triticale do not have a best match in *T. aestivum* with sequence identity higher than 90%, whereas 64% of random rye contigs have a best hit of 90% or higher in *T. aestivum* (Figure 1B). The number of rye gene hits in the wheat EST database was relatively modest, some 29 wheat ESTs; however, when these ESTs were compared back to the rye reference set, 62% had matches in the rye reference set with a percent identity higher than the identity of the initial match between the non-expressed rye genes and the wheat ESTs. Approximately one third of these ESTs had matches higher than 95% identity in the rye reference set, and approximately two thirds had a match with higher than 90% identity. The great majority of the reciprocal searches had matches that were characteristic of orthologous genes between rye and wheat (Additional file 6: Table S2). This indicates that the initial wheat ESTs hit by rye genes not expressed in triticale do not represent the homeologs of the rye non-expressed genes, and by extension, that the hits in the IWGSC-WSS database with lower than 90% identity are not homeologs of the rye genes (Additional file 6: Table S2). The lack of a homeologous copy in the A and B genomes of wheat for the rye genes not expressed in triticale would need to be confirmed with synteny information that is not yet available for these genomes on a scale wide enough to address this question. It is possible that the high degree of sequence similarity among homeologous gene copies for caleosin, α-tubulin, and Acc gene families may not be the case for other homeologous gene copies in triticale [5,27,34].

**Figure 2:**
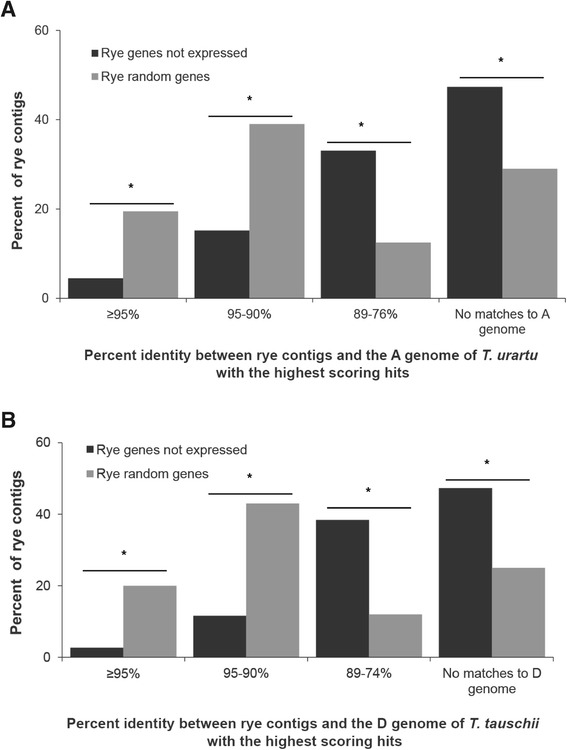
**Comparison of rye genes not expressed in triticale with the diploid genomes of** ***T. urartu*** **and** ***T. tauschii*** **.** Rye genes not expressed in triticale (black bars) have lower percent identity to their highest scoring hits in the genomes of **(A)** *T. urartu*, the ancestor of the A genome in *T. aestivum* and *T. turgidum*, and **(B)** *T. tauschii*, the ancestor of the D genome in *T. aestivum,* than to random rye contigs (gray bars). χ2 contingency tests were carried out to test if the number of rye genes not expressed triticale was significantly different from the number of random control rye genes in each class of percent identity to hits in the genomes of *T. urartu* and *T. tauschii.* *marks significant differences with p < 0.01 in the number of non-expressed rye contigs and random rye genes in each class of percent identities between rye contigs and A genome or D genome.

These results give rise to an empirical question: Do non-detected rye genes appear to lack a sequence in wheat with high similarity because the comparisons were biased by the comparison to the *T. aestivum* databases? The *T. aestivum* genome has experienced two allopolyploidizations; it is possible that genes might be selectively lost following polyploidization and thus were previously eliminated from the *T. aestivum* genome. In addition, the triticale analyzed here is derived from *T. turgidum*, which carries only the A and B genomes. The hexaploid *T. aestivum* was used as the primary basis of sequence comparison since the data sets available for *T. aestivum* are far larger than those for other *Triticum* species. To investigate this question, the same comparison between rye genes not expressed in triticale and the rye random set was performed with the draft genomes of two of the diploid progenitors of *T. aestivum*. The draft genome of *T. urartu*, the A genome donor, includes 499,222 scaffold assemblies [35], and *Ag. tauschii*, the D genome donor, includes 429,893 scaffold assemblies [36]; both are available in the GenBank NR database. Although both data sets have less depth than that of *T. aestivum*, the comparisons support the previous observations based on *T. aestivum*; only 19% and 14% of the non-detected rye genes had matches with 90% or higher sequence similarity in the A or D genome, respectively, whereas approximately 59% and 63% of the randomly selected rye control set of genes had matches with identities greater than 90% in the A and D genomes respectively (Figure 2A and B).

### Gene descriptions and ontologies of non-expressed rye sequences in triticale

A comparison of the 112 non-detected rye genes to the GenBank NR databases through the BLAST2GO workstation [32], resulted in 60 contigs with significant similarity to GenBank annotated protein sequences, tabulated in Additional file 3: Table S3; those without a match in the protein database are listed in Additional file 4: Table S4. The ontology of the non-expressed rye genes that were found by a BLASTx search in the GenBank NR database was varied, but the majority of these code for proteins with catalytic activity and proteins with nucleotide-binding and ion-binding activity (Additional file 3: Table S3). The BLASTx results revealed five rye disease resistance genes with an NB-ARC domain, a novel nucleotide-binding signaling motif shared by proteins encoded by plant disease resistance genes and regulators of cell death in animals. The NB-ARC domain-containing rye genes, namely R1, R8, R11, R19 and R20, were highly expressed in rye stem and their abundance ranged from 13.9 to 48.6 RPKM. The proteins most similar to R1, R8, R11, R19 and R20, in the GenBank NR database were encoded by putative disease resistance genes RGA1 (GB: EMT10593.1), and RGA3 (GB: EMT03843.1) from *Ag. tauschii*, and RPP13 from *T. urartu* (GB: EMS68441.1), and RPP13 from *Ag. tauschii* (GB: EMT01897.1). Plant resistance genes, R genes, have previously been reported to be eliminated by allopolyploidization; genomic analyses in Arabidopsis, cotton and soybean, indicated that these genes, especially Nucleotide Binding-Leucine Rich Repeat (NB-LRR) genes, were preferentially lost following polyploidization [37-39].

### Non-detected rye genes likely to be absent from triticale genome

**Figure 3:**
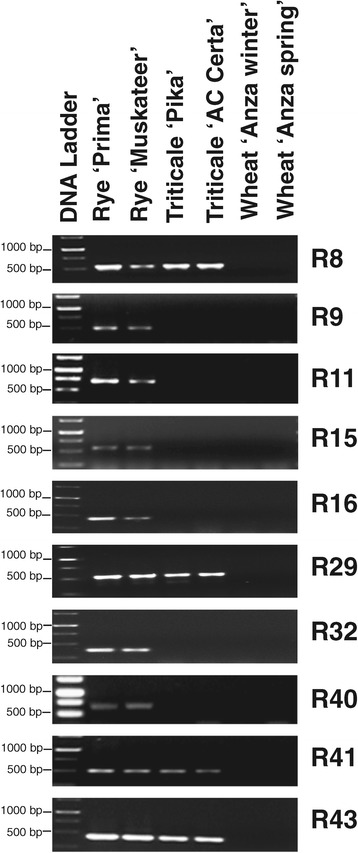
**PCR amplification of non-expressed rye genes in rye, triticale, and wheat.** A selected set of ten rye reference sequences that were not expressed in all of the triticale tissues tested were used to screen by PCR amplification in the genomic DNA of triticale for evidence for the absence of genes using genomic DNA of two cultivars of rye, triticale, and wheat plants. The molecular size ladder has DNA fragments of molecular sizes of 1500 bp, 1000 bp, 750 bp, and 500 bp.

**Figure 4:**
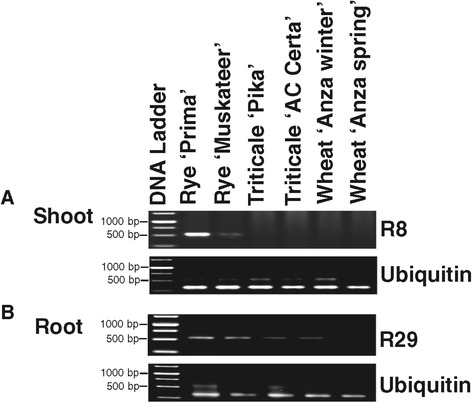
**Validation of the silencing of two rye genes in triticale.** Two rye coding sequences that were not expressed in triticale but found to be present in the genomic DNA of triticale were rescreened by RT-PCR on the cDNA generated from RNAs of two-week old rye, triticale and wheat seedling. Ubiquitin gene was used as a control for the expression in all plants. **(A)** RT-PCR revealed that R8 from the rye genome was silenced in triticale shoot. (**B)** Low expression of R29 from the rye genome was detected in triticale root. The ladder has DNA fragments with molecular sizes of 1500 bp, 1000 bp, 750 bp, and 500 bp.

The lack of rye gene expression in the triticale background could have been due to the absence of the gene. To verify this, a PCR assay was performed using primer sets derived from ten rye genes that were not detected in the survey of the 454-expression profile. Six out of ten rye candidate genes were found to be absent from the triticale genomes, although they were present in the rye cultivars (Figure 3). Wheat cultivars showed no amplification products, as expected, since closely matching sequences were not found in wheat genome assemblies. The six rye genes, R9, R11, R15, R16, R32 and R40, were absent from the genome of both triticale cultivars. Four genes R8, R29, R41 and R43 were detected in the triticale genomic DNA. Two of these were re-assessed for expression by RT-PCR analysis in the same triticale and rye cultivars used to initially identify the candidate silent genes. The analysis did not detect any expression from R8 in the cDNA generated from two-week old triticale plants, although the expression of the same gene was found in the rye shoots. However, the expression of R29 was detected at low levels in triticale and rye roots (Figure 4). The other two silent genes detected in the genomic DNA of triticale, R41 and R43, were not assayed by RT-PCR; they were initially detected in rye anther 454 cDNA.

### Potential mechanism for genetic alteration of allopolyploids

The combination of diverged genomes in newly formed allopolyploids can result in dramatic changes in the genome structure and in the transcriptome. Allopolyploidization results in chromosome loss [1], gene loss [2,6,7], gene silencing [8] and altered gene expression [3]. It has been suggested that these changes occur under extreme selection for the formation of stable fertile hybrids, and changes in genomes formed in allopolyploids likely increase fitness [15]. Both triticale and wheat have high degrees of plasticity due to their natural capabilities to overcome such dramatic changes in both gene expression and genomic restructuring [10,40]. The structural genomic changes might play a vital role in chromosome pairing during meiosis, restoring the full fertility of the plant after the extreme genetic shock faced by the new hybrid. Although this process occurs naturally, genome changes do not appear to be random [8,40]. In the current study, most of the rye silenced genes in triticale apparently have low sequence similarity to genes in the other genomes of triticale. Of the ten rye genes that were not expressed and were selected to test for their presence in the genome of triticale, six were found to be absent. The rye genes absent or unexpressed in triticale could be accounted for by gene deletion after polyploidization or by their absence in the rye lines that were parental to the triticale lines used in the study. The pedigrees of triticale cultivars are complex with multiple sources of the rye genome and the rye cultivar lines analyzed here are not the direct parental lines of these triticale cultivars. In either explanation of gene loss, genes with lower sequence conservation are also less conserved by the presence/absence criteria. Previous studies showed that partial genome elimination after triticale polyploidization was biased for elimination from the rye genome. It appears that there are molecular mechanisms for recognition and elimination of sequences that are dissimilar to one of the parental genomes in this set. Ma and Gustafson demonstrated that the rye genome undergoes substantially more genomic reorganization and changes in gene expression compared to the A and B genomes in order to adapt to a triticale background, as it was found in AFLP analyses that 65% of rye bands were lost in triticale compared to only approximately 20% of bands for wheat [41]. Though numerous deletions were detected in wheat and triticale hybrids by AFLP analysis, this analysis did not differentiate between regions that had high or low sequence similarity between the two parental species, nor did the analysis distinguish between coding and non-coding regions [40,42]. Similarly, the analysis of newly formed triticale [23], allotetraploid wheat [8] and allohexaploid wheat [6,40] by Restriction Fragment Length Polymorphism analysis, revealed coding regions that were deleted from the A, B, D or R genomes. However the probes cross hybridized to several genomes and would not detect sequences in one sub-genome that did not share high sequence similarity with another sub-genome. The high degree of DNA loss, on the order of 20%, from the R genome, reported in studies that surveyed the whole genome [19,21], compared to the loss of expression of approximately 1% of genes surveyed in this study suggests different mechanisms for DNA loss for coding and non-coding regions. The analysis of the well characterized hardness locus, (*Ha*) locus, gives insight into the susceptibility of particular regions of the genome for elimination. The DNA sequence of this locus which regulates seed hardness in wheat indicated that regions of the genome may be particularly liable to deletion [43,44]. Though selection in agriculture is clearly a strong driving force for the preservation of deletions leading to hard seeds, a comparison of allotetraploid and allohexaploid wheat showed that the alleles arose several times independently. What is particularly revealing is that the breakpoints for deletion were very similar, but not identical; such a pattern indicated a bias or targeting of this region for deletion, and the authors suggested that they may be related to transposon activation and illegitimate recombination [43]. Although great efforts have been made to detect the genetic changes and epigenetic modifications subsequent to genome hybridization and doubling, understanding gene regulation mechanisms by merging two or more genomes was not an easy task. The investigations have implicated several mechanisms, including intergenomic recombination [45], transposon activation [46,47] and double-strand break repair [45]. Studies in yeast showed that the presence of unpaired regions of DNA within homologous sequences triggers mismatch repair proteins to correct sequence DNA differences [48,49]. The repair system involves DNA strand invasion between the heterologous sequences with deletion of unpaired sequences. The example of heterologous sequence elimination through deleting unpaired loops was based on pairing between homologous chromosomes. Could this process occur between homeologous chromosomes? Though homeologous recombination is strongly repressed in polyploid wheat by the *Ph1* locus, an invasion of the A genome by sequences from the B genome was identified in the tetraploid wheat using genomic *in situ* hybridization [50,51]. Comai [22] reported that homeolog paring can lead to chromosomal deletion, resulting in the breakdown of the post-replicative mismatch repair system. The excessive increase in the potential for mismatches from strand invasion between homeologous chromosomes could lead to saturation and dysfunction of the mismatch repair proteins that normally have roles in blocking homeolog recombination. The rye genes absent in triticale detected in this analysis of high-throughput sequencing offer important candidate genes for further analysis. The comparison of BAC clones for these genes from rye and triticale would help to better understand the nature and extent of these potential deletions, especially if they were relatively small and flanking regions could be identified in clones from both rye and triticale.

## Conclusion

The analysis of second generation sequence data derived from mRNA from rye and triticale revealed that approximately 0.5% of genes which were relatively highly expressed in rye were not detected in triticale. A survey of ten of these genes indicated that six were absent from the genome of the triticale cultivars analyzed here. These genes may have been deleted after the polyploidization events that gave rise to these triticales or they may have been absent in the rye progenitor lines of the triticales. A striking feature of this set of genes is that they have markedly low degree of sequence similarity to their most similar wheat genes. The results suggest that genes that are more likely to be deleted in the Triticeae genomes are those that do not have homeologous copies in the polyploid genomes. Further genomic studies in rye and triticale are necessary to detect the recombination motifs at the sites of deletion, and to decipher the mechanisms of genome rearrangement and evolution.

### Availability of supporting data

The sequence of the 112 rye genes not expressed in triticale are included as Additional file 7. The Roche 454-cDNA sequence libraries for *Secale cereale* are deposited at DNA Data Bank of Japan with identifier DRA000384 and are available at NCBI, Sequence Read Archive, with study identifier DRP000390, BioProject PRJDB2278 and accessions numbers DRX000652 to DRX000659. The Roche 454-cDNA sequence data sets for triticale are deposited at NCBI with study identifier SRP055516, BioProject PRJNA276398, and run identifiers SRR1818724, SRR1819193, SRR1819817 to SRR1819824, and SRR1821191 to SRR1821204. The Transcriptome Shotgun Assembly project for *Secale cereale* has been deposited at DDBJ/EMBL/GenBank under the accession GCJW00000000.

## Additional files

Additional file 1: Table S1. Primers used to study the presence or absence of rye genes from R genome in triticale. Additional file 2: Figure S1. Flowchart used to detect silent rye genes in triticale. Additional file 3: Table S3. Annotation of rye genes silenced in triticale. Additional file 4: Table S4. Rye silenced genes in triticale that did not have any similar match to GenBank NR database based on BLAST2GO with minimum E-value of e-10. Additional file 5: Table S5. Percent identity of silenced rye genes with IWGSC Triticum genomic seq. Additional file 6: Table S2. The percent identity of silenced rye genes in reciprocal searches. Additional file 7:
**Full set of 112 rye gene sequences not expressed in triticale with Contig IDs.**

## Abbreviations

IWGSC-WSS: International Wheat Genome Sequencing Consortium-Wheat Survey Sequencing
RPKM: Reads per kb of gene length per million
χ2: Chi Squared
