# Supplementary material for: Identification and characterization of rye genes not expressed in allohexaploid triticale
Source: BMC Genomics. 2015 Apr 10;16(1):281. doi: 10.1186/s12864-015-1480-x (PMC4396786; doi:10.1186/s12864-015-1480-x)
Supplement: Additional file 2: Figure S1. — Flowchart used to detect silent rye genes in triticale. [file 12864_2015_1480_MOESM2_ESM.pdf]

Additional file 2

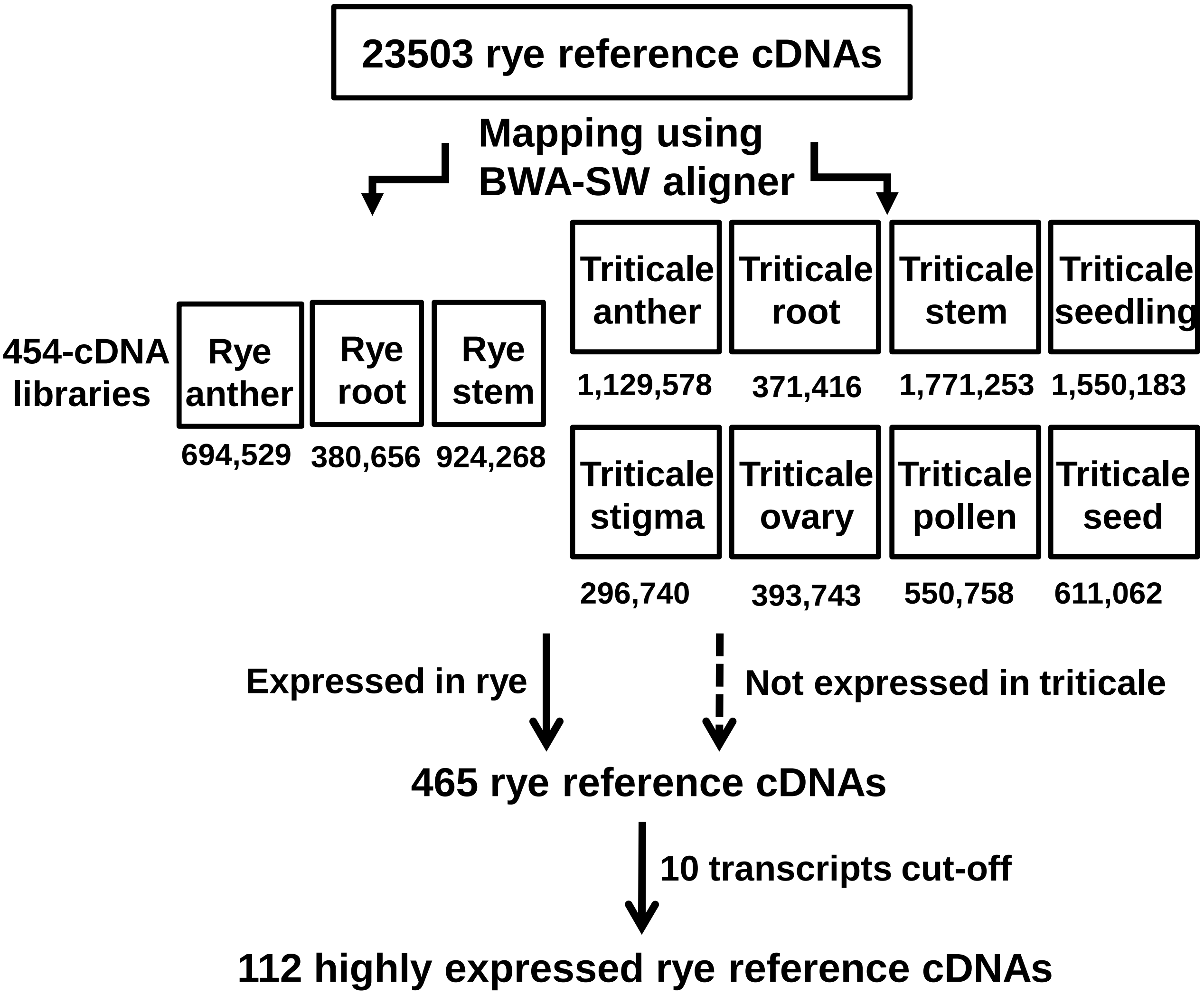

Figure S1. Flowchart used to detect rye genes not expressed in triticale.
RNA-seq workflow was performed to characterize the expression of 23,503 rye reference contig assemblies. Rye and triticale transcripts were aligned to their corresponding rye reference assemblies using BWA-SW algorithm of BWA. About 465 reference sequences were found to be expressed in at least one rye tissue and were not found in any of the eight triticale libraries used in the study. Out of these, 112 rye reference sequences were found to be not expressed in all triticale tissues. The depth of each library (the number of transcripts in each library) is written underneath each tissue.
